# Tower of Belém (Lisbon)–Status Quo 3D Documentation and Material Origin Determination

**DOI:** 10.3390/s20082355

**Published:** 2020-04-21

**Authors:** Paula Redweik, José Juan de Sanjosé Blasco, Manuel Sánchez-Fernández, Alan D. Atkinson, Luís Francisco Martínez Corrales

**Affiliations:** 1 Department of Geographic Engineering, Geophysics and Energy, Faculdade de Ciências, Instituto Dom Luiz, Universidade de Lisboa, Campo Grande, 1749-016 Lisboa, Portugal; 2 NEXUS Research Group, INTERRA Research Institute, University of Extremadura, 10003 Cáceres, Spain; jjblasco@unex.es (J.J.d.S.B.); msf@unex.es (M.S.-F.); atkinson@unex.es (A.D.A.); luisfrancisco@unex.es (L.F.M.C.)

**Keywords:** Cultural heritage, geomatic techniques, 3D model, spectral signature, quarry

## Abstract

The Tower of Belém, an early 16th century defense tower located at the mouth of the Tagus river, is the iconic symbol of Lisbon. It belongs to the Belém complex, classified since 1983 as a World Heritage Site by the UNESCO, and it is the second most visited monument in Portugal. On November 1st, 1755, there was a heavy earthquake in Lisbon followed by a tsunami, causing between 60,000 and 100,000 deaths. There is a possibility of a repetition of such a catastrophe, which could bring about the collapse of the structure. This was the reasoning behind the decision to evaluate the Tower of Belém by means of surveys using Terrestrial Laser Scanning and photogrammetry. Until now, there was no high-resolution 3D model of the interior and exterior of the tower. A complete 3D documentation of the state of the Tower was achieved with a cloud of more than 6,200 million 3D points in the ETRS89 PT-TM06 coordinate system. Additionally, measurements were made using a hyperspectral camera and a spectroradiometer to characterize the stone material used in the Tower. The result is a digital 3D representation of the Tower of Belém, and the identification of the quarries that may have been used to extract its stone. The work carried out combines geometrical and material analysis. The methods used may constitute a guide when documenting and intervening in similar heritage elements. Finally, the information contained therein will allow an eventual reconstruction of the Tower in the case of another catastrophe.

## 1. Introduction

The Tower of Belém is one of the most famous and most visited monuments in Lisbon and throughout Portugal. It is highly representative of the era of Portuguese maritime discoveries and commerce. It was built during the reign of Manuel I (1495–1521) following the earlier plans of Joao II (1481–1495) to protect the entrance to the city of Lisbon through the Tagus Bay [1]. The tower was built (1514–1520) by Francisco de Arruda under the direction of Diego Boytac (the Royal Building Master), who also worked on the construction of the Hieronymites Monastery [1].

**Figure 1:**
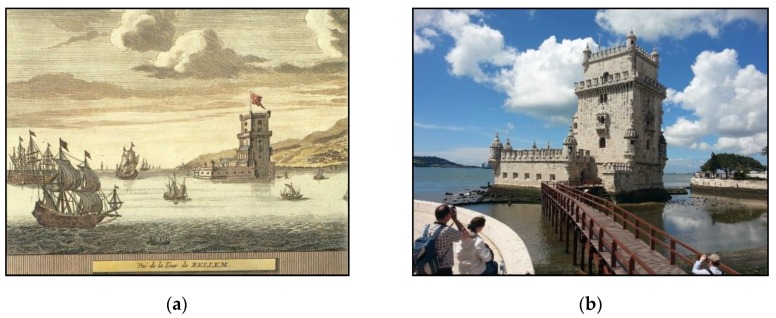
(**a**) Old illustration of the Tower of Belém [1]; (**b**) The tower as it stands today.

The Tower of Belém is the iconic symbol of the city of Lisbon and a memory of Portugal’s golden age of discoveries (Figure 1a). It consists of a fortified tower built on a rocky outcrop on the Tagus river not far from the north riverbank. The tower was planned as a defensive position at the entrance to Lisbon harbour together with two other fortresses on land, in Trafaria, on the opposite side of the river, and Cascais, 18 km to the west, and with some armed ships (caravels) placed on the Tagus [1].

A pentagonal bulwark and a gothic tower are the main architectural features of the building, and these contain several elements of the Manueline style, the Portuguese renaissance, such as ropes, armillary spheres and shield-shaped battlements emblazoning the Cross of the Order of Christ (Figure 1b). Due to its historical and architectonic significance, the Tower of Belém was included in the UNESCO World Heritage List in 1983 together with the nearby Hieronymites Monastery [2,3]. The building consists of two floors in the bulwark, one of them below the water level, and five floors in the tower over a large cistern, which was used to collect rain from the upper terrace to ensure a fresh water supply. The whole monument was built from Lioz limestone, a rare kind of white limestone found in Lisbon and surrounding area.

In 1992, the Exterior Conservation Project of the Tower of Belém was undertaken by the World Monuments Fund (WMF) and its Portuguese affiliate (WMF Portugal) [1,4]. Its state of conservation was analysed and some preliminary tests were carried out for cleaning and replacing the mortar. The documentation of the state of conservation of the exterior of the tower served as the basis for the intervention and subsequent restoration. A detailed topographic survey was carried out, allowing the creation of a graphic database on which to photograph, identify, measure and quantify the different pathologies of the monument. It is important to notice that only a topographic survey of the exterior of the tower was made [1,3,4,5]. After an exhaustive analysis in collaboration with the Portuguese Institute of Architectural Heritage, the conservation project was completed in January 1998. The project was carried out in two different phases (one for the tower and the other for the bulwark), and these interventions can be divided into four categories: structural interventions (misaligned and deteriorated elements), treatment of joints (due to water penetration through horizontal surfaces, open joints, larger holes between blocks, and cracked or fissured blocks), cleaning (surfaces soiled by air pollution and/or biological colonization) and other specific treatments. In 1999 these works received the Europa Nostra Award from the European Association for the Protection and Enhancement of Europe’s Architectural and Natural Heritage [4]. Half a decade later a new analysis of the results of the restoration was carried out, offering highly satisfactory results regarding the durability of the intervention [5]. Previous documentation and that produced during the restoration project must serve as the basis for the detection of possible changes over time, as will the documentation produced in the present project.

The earthquake of 1755, together with fire and the subsequent tsunami, devastated the city of Lisbon [6,7,8]. Since then, numerous studies have been conducted to try to quantify the true magnitude of the disaster in the city [9]. Today, some models estimate that a similar tsunami could reach a maximum flooding extent of 1.29 km into the Alcantara valley and depths of close to 10 m in some places in the city of Lisbon [10]. In view of this event, public and scientific institutions have made efforts to quantify risks in the possibly affected areas as accurately as possible so that the population can be alerted to the risk and adequate evacuation plans can be designed [11,12,13,14].

Due to its location in the Tagus river estuary, the integrity of the Tower of Belém could be affected by an earthquake and/or tsunami similar to those that happened in 1755. Given this situation, a detailed inventory of the state of the monument is very much needed. Geomatic techniques for the elaboration of high resolution 3D models of this kind of architectural construction are a very useful tool for making an inventory of historical heritage [15]. 3D techniques have become an indispensable tool for the structural analysis of important architectural monuments. They provide the 3D documentary information that forms the basis for subsequent studies of conservation, rehabilitation and reconstruction of such monuments [15,16,17]. Non-destructive techniques for the study of historical heritage offer great advantages to the task of restoration [18].

Massive data acquisition techniques based on photogrammetry or on the use of LiDAR, Terrestrial Laser Scanner (TLS), changed the way to approach heritage documentation projects, architectural surveys for the elaboration of intervention and conservation projects or structural evaluation studies based on geometric variations [19,20,21,22,23]. When addressing a project it is important to consider which of the existing techniques is the most appropriate [24]. The precision required for the final product conditions the technique and method to be used in the survey. There are several research works which compare close-range photogrammetry and TLS for heritage documentation in diverse study cases [23,24,25,26,27]. Some papers indicate similar precisions obtained by both techniques, and conclude that the principal difference between them are the characteristics of data acquisition [23,24].

In [28], different cases of studies are detailed in which there is a need to document with a high level of detail heritage entities for conservation or intervention, but the level of detail is not quantified, only the nature of the elements and the final objective. In [22], the working process developed for a specific case study is qualitatively documented, whose objective is to obtain orthoimages from colored point clouds and 2D CAD planimetry, and where close-range photogrammetry and TLS are used for its documentation. Other authors base the definition of the level of detail of the necessary documentation based on the concept of Level of Development (LOD) extracted from Building Information Modelling (BIM) working methodology [29]. The need to know the geometry in detail of the masonry structures of the building to be able to estimate the state of structural health is described. Close-range photogrammetry compared to TLS requires the use of control points to be able to perform the scaling and georeferencing of the model. This condition is one of the important differences when applying the working method. In [30] this need is revealed and a methodology is proposed to be followed to solve the problem. The integration of models obtained by different techniques (close-range photogrammetry and TLS) can be carried out by different methodologies. In [30], the architectural survey is carried out by both techniques and the integration of 3D models is solved using common control points and the establishment of a single coordinate system. The authors of [31] resort to the integration of close-range photogrammetry and TLS as documentation techniques for the drafting of a project of integral recovery of the building as well as its correct historical interpretation and construction phases. In the case of [32], the authors consider the 3D documentation of the element necessary to perform a correct archaeological analysis, using close-range photogrammetry and TLS as an architectural survey tool.

In the present project, in order to document the actual state of the monument, an as-built detailed 3D survey of the interior rooms and exterior walls was conducted. In the initial approach a terrestrial laser scanner was the only equipment considered for the work, but the proposal to station the scanner on the surrounding rocks during the hours of low tide in order to survey the south walls of the bulwark was very soon ruled out as being too risky for personnel and equipment. A photogrammetric survey using a vessel as a platform for the camera was the preferred solution for completing the primary data collection.

**Figure 2:**
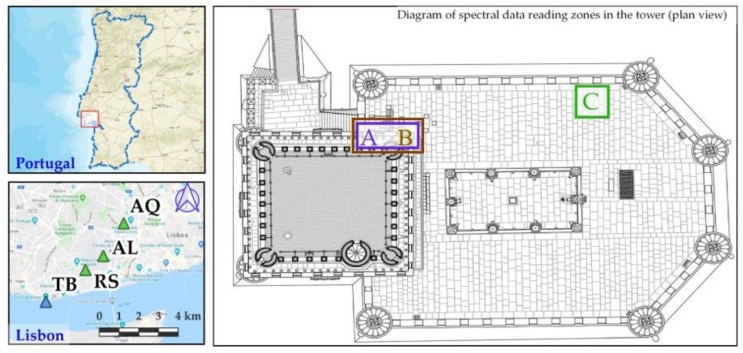
(Left) Location of the Tower of Belém (TB) and of the old Lioz quarries of Rio Seco (RS), Alvito (AL) and Aqueduto das Águas Livres (AQ). (Right) Location of spectral data reading zones in the Tower: A (purple) data acquisition of original Lioz with hyperspectral camera. B (brown) data acquisition of original Lioz with spectroradiometer. C (green) data acquisition of silica (dark spots) with spectroradiometer in an ashlar of the Tower.

In order to find the possible origin of the ashlars used to build the Tower of Belém, the most likely quarries were first located with the aid of a geologist. The criteria used were the following: the quarry had to be of Lioz, the kind of limestone used in the tower; the probable use of the quarry had to be old enough to have already been exploited in the 16^th^ century; it could not be too far away from the tower since transportation of the stones was not an easy task; and the last criterion was one of a practical nature: the quarry face had to be accessible to the measuring instruments and not filled in with foreign matter. Three possible sites were chosen: Rio Seco, now an urban park, Alvito, an abandoned area, and Aqueduto das Águas Livres, an accessible area near a highway access (Figure 2). The latter was situated further away, but the fact that there had been a river feeding into the Tagus near the quarry until 1945, when it was channelled underground, raised the possibility that the stones could have been transported southwards to the Tagus by boat and from there westwards to the site of the tower all the way over water. Based on the established geological and historical context, molecular absorption spectrometry techniques were used to analyse the material in the different locations, as well as certain ashlars of the Tower of Belém. Hyperspectral instruments and a spectroradiometer were applied to carry out this identification task. With the recorded data, a comparison of the absorption values obtained in the different locations was made. This work allows establishing the similarity between the compared materials from a qualitative analysis of the results. The different spectrometry techniques are focused on the analysis of the composition of materials. Portable equipment such as hyperspectral cameras, RX equipment or spectroradiometers, make it possible to perform analysis of this nature without the need to extract a sample for laboratory analysis [33,34,35]. Working methods with the spectral techniques applied to heritage are described in [33]. Works of art are catalogued. For the cataloging of these, given the size and manageability, the analysis of spectral nature in order to establish similarities and differences between the pigments of different areas of the same work, has been of greater application [33,36,37]. For buildings, the use of these techniques is focused on archaeological and constructive studies as shown in [34,35]. In these works, the origin or composition of materials are determined from the spectral analysis differences, so that construction times or pathological conditions can be established.

The works carried out in the Tower of Belém have a main objective: to make available to the conservation and intervention tasks in the monument an exhaustive 3D representation from which 3D models, plants and elevations can be made at ease, and the location of materials that have the same origin. These contributions allow the monument management a greater knowledge of the building so that intervention tasks can be organized more efficiently.

The characterization of the monument from the geometric point of view through an exhaustive and accurate 3D representation allows future studies of degradation of the monument, evolution of pathologies or structural health. On the other hand, it is sought to establish the origin of the materials used for the construction of the Tower so that any present or future alteration in the construction can be repaired with materials with the same characteristics. The study carried out proposes a methodology that solves the problems when it comes to acquiring geometric data or determining the origin of materials of a monument as singular as the Tower of Belém.

## 2. Methods

The acquisition of 3D data of the Tower of Belém is a photogrammetric and TLS surveying challenge for several reasons. The tower is currently in use as a tourist attraction, being the second most visited monument in the country. A great many people access the building over a visitable area of about 1500 m^2^, which means that surveying work can only take place on Mondays, the day the tower remains closed to the public for maintenance. Another problem is that there is furniture in the building that is not fixed, particularly on the entrance floor. While surveying the building, any presence of furniture can obstruct the sight to relevant architectural elements or provoke scanning shadows when it is near the scanner. Scans had to be performed after removing most of the furniture. Nevertheless, the battery of cannons in one of the floors, had to remain in place due to their weight. This fact did not affect cloud registering but caused some occlusions near the openings where the cannons were placed, which, nevertheless, were considered not significant in the overall coverage. Finally, the geometric configuration of the tower and situation in the Tagus river, together with the availability of only one day a week for surveys, constrained the surveying works.

For the 3D surveying of the building, a terrestrial laser scanner (TLS) was used, but for the most complicated areas, close-range MultiViewStereo photogrammetry was the chosen solution. The south side of the tower, involved by water, was surveyed with a photographic camera from a vessel. Photogrammetry was used as well for the spiral stairs connecting the different levels inside the Tower of Belém. The Global Navigation Satellite System (GNSS) was used for registering the 3D point clouds, both from TLS and from photogrammetry. The reference coordinate system established in the work was ETRS89 PT-TM06, the official reference system in Portugal [38].

For the spectral definition of the ashlars used in the construction of the tower, a spectroradiometer and a hyperspectral camera were used. These instruments were applied to selected ashlars of the Tower of Belém and in the quarries nearby from which it is reasonable to assume the stone for the ashlars was extracted. Using the information obtained by the hyperspectral camera, a supervised classification was made by means of training a neural network in order to identify the different types of rocks at the quarry faces, thus delimiting the areas to survey using the spectroradiometer on the ashlars of the Tower of Belém.

The characterization and analysis of rock materials through reflection spectrometry allows heritage to be studied non-destructively [39]. The use of neural computing in remote sensing is useful as it delimits the physical frame from which to gather data from a material, in this case Lioz microcrystalline limestone. It was this kind of limestone that was used in the construction of the Tower of Belém [40,41].

**Figure 3:**
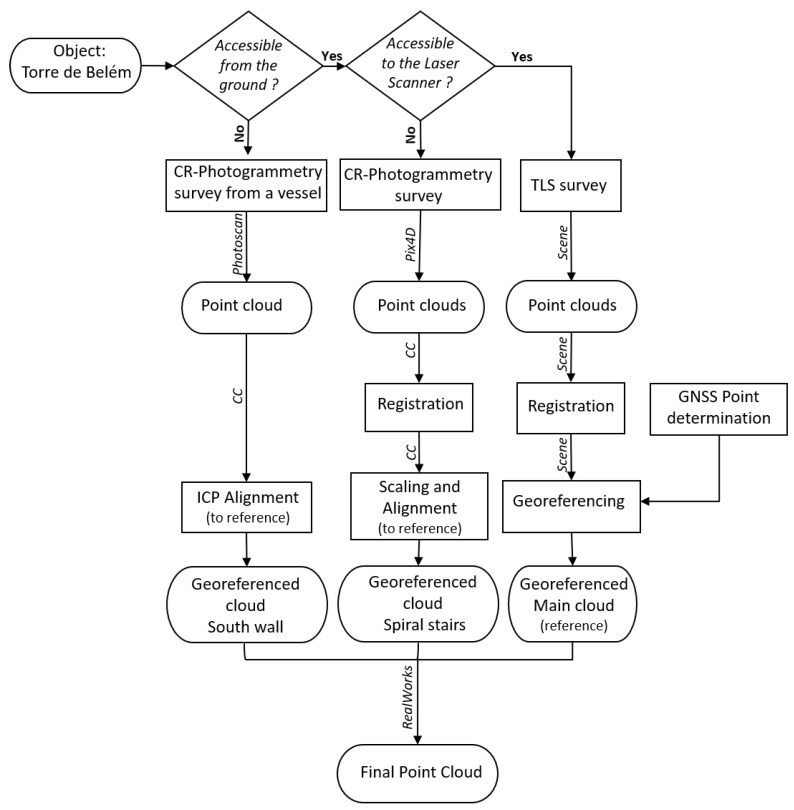
Work methodology applied in the geometric characterization. CC–CloudCompare [42]; Photoscan–Agisoft Photoscan [43]; Pix4D–Pix4D Mapper [44]; Realworks–Trimble RealWorks [45]; Scene–Faro Scene [46].

Figure 3 shows in a schematic way the workflow and the software used for the geometric description of the Tower of Belém. The terrestrial laser scanner was used to acquire data on the geometry of the exterior of the tower accessible from the ground and of the interior of the tower accessible to the scanner. The purpose of the 3D survey is the documentation of a heritage building, this requiring a distribution of 3D scans that allow covering as much of the building as possible. The distribution of scans is also conditioned by the methodology used for point cloud (scan) register and the resolution/scanning time ratio that the configurations of the used scanner allow. In cases where it is not possible to use TLS, close-range photogrammetry was used.

The spiral staircase was reconstructed photogrammetrically from images taken from inside as described in Section 3.1.2. Its scaling and georeferencing is obtained by an alignment with the georeferenced TLS point clouds with corresponding conspicuous point pairs manually measured in both clouds. No marks were used due to monument restrictions.

For the photogrammetric model of the south façade, images were taken from a vessel at a mean distance of 55 to 60 m from the tower south wall. The camera was handheld, and the acquisition geometry was conditioned by the maritime and meteorological conditions at the acquisition time. Shutter speed, aperture and ISO (light sensibility) were chosen during the first of the three shot sequences in order to obtain the less influence of the vessel movement and an optimal image from the radiometric point of view.

To establish the coordinate system, static measurements in several points were made with simultaneous GNSS systems. The determined points were defined as check and control points for the georeferencing of the 3D point clouds. In the case of point measurements on vertical faces, a total station and the radiation method were used. For the bearing determination, points determined with GNSS at greater distances were used.

The geometric models of the point clouds of the referred sources (close-range photogrammetry and TLS) were all displayed simultaneously in the Trimble RealWorks software [45]. Although they are not merged in one cloud, this makes possible to work with a complete 3D representation of the building, because all points are in the same global coordinate system. Graphical products (3D models, elevations from vertical cuts, plants from horizontal cuts, other mappings) can be derived from this representation.

As for the evaluation of materials, it started with the global visual inspection of the element and proposed quarries. Then, the spectral evaluation of specific or singular elements of the tower and quarries was carried out and results were compared to establish the origin of the studied materials. The visual evaluation of the quarries was preceded by a geological search and consultation of the surroundings of the Belém neighborhood of Lisbon. A geological interpretation of the quarries and ashlars of the tower was necessary to design the spectral survey. This was carried out using two main techniques: data collection with a spectroradiometer, that allows the spectral signature of the analysed materials to be extracted, and the use of the hyperspectral camera that allows the classification of the elements contained in the surveyed scenes. This operation certifies the similarity of materials contained in the scene.

## 3. Data Acquisition and Processing

### 3.1. Close-Range Photogrammetry

#### 3.1.1. Exterior Walls (South)

A challenge to the project was posed by the fact the south side of the bulwark is facing the river, and cannot be observed from land. Surveying the south exterior wall with a TLS would mean having to station the laser scanner on the muddy and slippery rocky riverbed at low tide. Besides being dangerous for both equipment and operator, the stationing distance to the wall would also be too short, leading to excessively inclined lines of sight at the periphery of the point cloud, since the outcrop around the tower is just a few meters wide. Stationing the scanner on a vessel would have necessitated a Global Navigation Satellite System/Inertial Measuring Unit (GNSS/IMU) coupled to the scanner for continuous orientation, but this equipment was not available.

**Figure 4:**
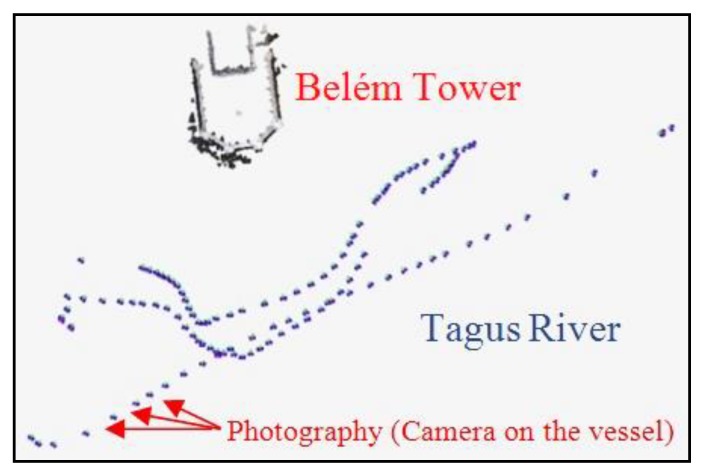
Scheme of camera positions on the vessel relative to the tower. Blue points indicate camera positions.

The easiest and most affordable solution was to use a small vessel in motion passing by the bulwark while photographing the tower. A handheld Sony α230 camera was used with a fixed focal length of 55 mm. A total of 119 overlapping photographs in three different almost horizontal sequences (Figure 4) were taken with the vessel moving slowly at constant speed and the perspective between adjacent images changing only slightly in order to guarantee a successful correlation later in the process. A cloudy day and the river almost free of waves contributed to the success of the survey. A rough water surface or a sunny day would have adversely affected the task, the former disturbing the correlation of the different photographs and the latter affecting the visibility of details in the images, since the white limestone of the tower would have reflected too much sunlight.

In order to generate the 3D point cloud of the south wall of the bulwark, the 119 images were processed using the software Agisoft Photoscan [43], which uses a modified version of the Scale Invariant Feature Transform (SIFT) algorithm for automatic detection of points of interest in each image and correlates several images through the similarity of the descriptors of the points of interest. An automatic calibration of the camera was used. The result is the relative orientation of all images and the spatial orientation of the whole set in a local reference system. A dense point cloud was then calculated by spatial intersection of detected homologous points. The dense point cloud describes the object in 3D. A 3D model with a continuous surface can be calculated from this point cloud. In this project only the point cloud was required since it was intended to produce plants and elevations and the dense cloud has all the geometric information needed for that task.

The quality of the relative orientation is expressed through the reprojection error. For the reconstruction of the south side of the Tower of Belém, two relative orientations were made using different start conditions. In a first orientation all the raw images were processed presenting an RMS value of reprojection error of 0.54 pixels with a maximum value of 15.90 pixels. Prior to the second processing, masks were constructed in each image hiding the areas of sky and dark pixels where the processing algorithm can fail, as well as all information outside the tower (trees, landscape, buildings, etc.). When performing a second bundle adjustment, reprojection values of RMS 0.39 pixels and a maximum of 8.18 pixels were obtained. The geometric quality of the final model is determined further by the method used for georeferencing. As mentioned in Section 3.3., control points were obtained either from the TLS point cloud of common areas or from direct measurement through two radiations made with a reflectorless total station. Radiations were supported by the same network of GNSS determined points used in the georeferencing of the TLS clouds. A third method for georeferencing the cloud was the adjustment to the TLS cloud using the Iterative Closest Point (ICP) algorithm implemented in the CloudCompare software [42].

**Figure 5:**
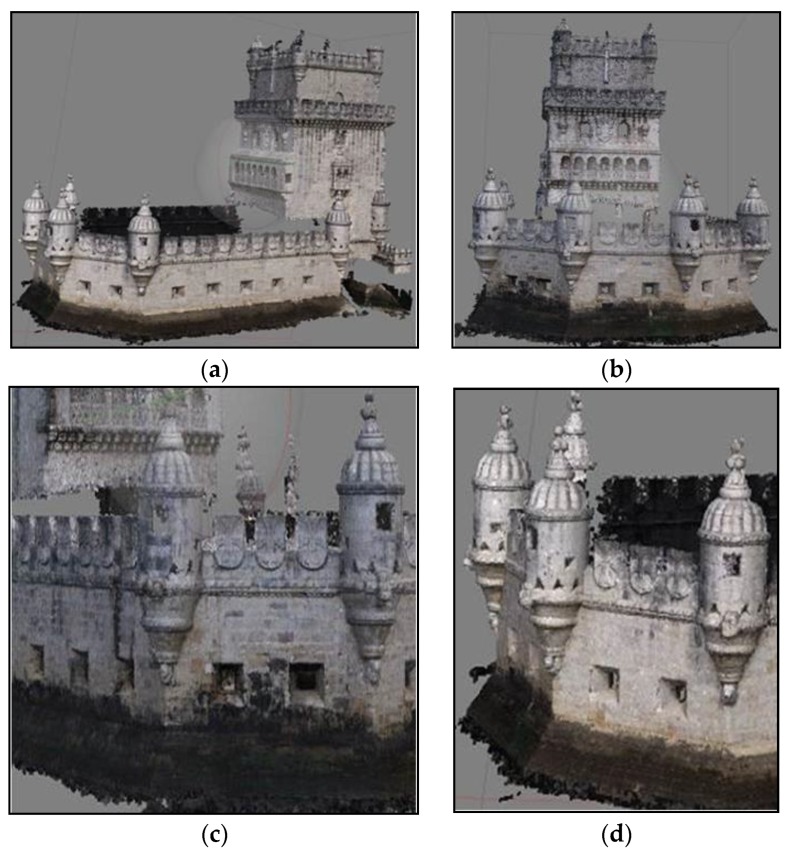
Photogrammetric 3D point cloud: (**a**) East wall; (**b**) South wall; (**c**) and (**d**) Details of the South wall.

A mean density of 5000 points/m^2^ was achieved for the exterior south walls (Figure 5), allowing individual stones and architectural details to be distinguished. Although it appears in the point cloud, the high tower itself will not be used in the final products since it had already been surveyed by laser scanning. The east and west walls were common to the laser scanned clouds and were used for georeferencing.

#### 3.1.2. Interior Stairs of the Tower

**Figure 6:**
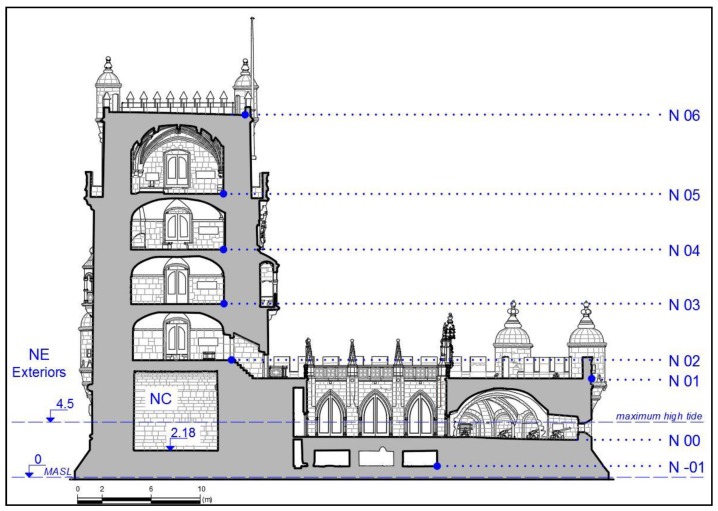
Levels surveyed in the Tower of Belém.

The levels inside the tower N02 to N05 (Figure 6) and the upper terrace, N06, are connected by very narrow spiral stairs. Spiral stairs are a complex object to survey due to the limited field of view from any point therein. 

**Figure 7:**
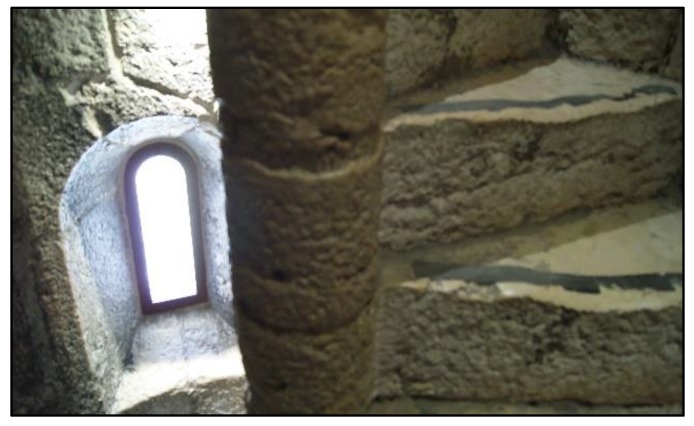
Spiral stairs of the Tower of Belém.

The dimensions of these stairs made it impossible, or at least very time-consuming, to survey them using a TLS. The stairs consist of 92 steps developing around a central column (Figure 7). The step width from the central column to the wall varied between 0.76 m and 0.79 m, just over the shortest distance that could be recorded by the laser scanner (0.60 m).

Neither were conditions very favorable for a photogrammetric survey. The stairs form a typical helical surface by winding around themselves but are enclosed in a dark vertical cylinder with only a few embrasures (five) and a door to each floor. Between two neighboring floors the stairs make two full circle turns and there is no distinction between stair flights. 

Nevertheless, a systematic photogrammetric survey was undertaken using the same camera as for the exterior south wall, a Nikon α230, but the fixed focal length was changed to 18 mm in order to gain a wider field of view.

The whole stairs were divided into four sections, each corresponding to a flight between floors. Various attempts were made to get suitable sequences of only slightly changing perspectives with adequate brightness and sharpness in such a sparsely illuminated space at short distances. The camera had to be handheld. Working stepwise (literally, step by step), more than one thousand photographs were taken per section, while two different approaches were followed during photo acquisition:

Four different sequences: steps, ceiling, central column and wall, with high overlapping.

**Figure 8:**
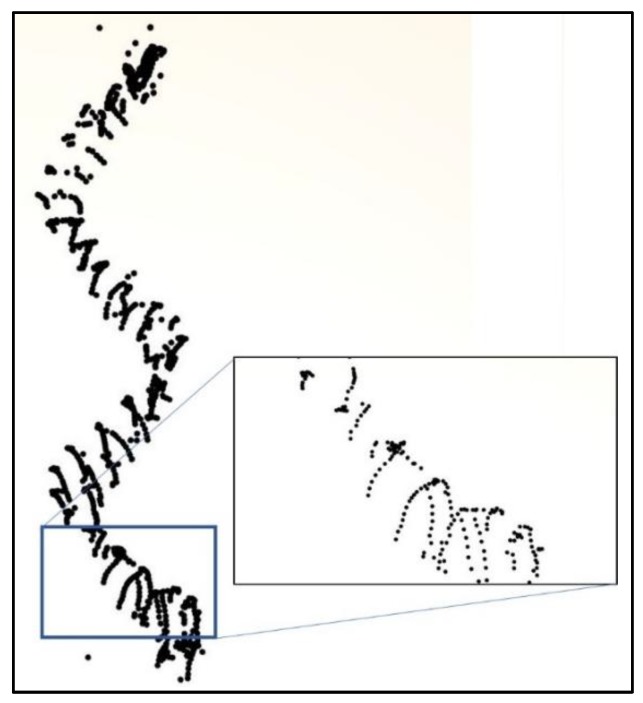
Camera stations (black dots) from the survey of section N04-N05 of the spiral stairs.

One sequence obtained with the camera following a spiral line along the stairs sweeping all surfaces continuously (step - wall–ceiling–column–next step–wall–ceiling–column–next step, etc.) (Figure 8).

A fisheye lens was not available as recommended [47,48], so work had to be done with the available 18 mm lens. 

The door connections on each floor were also surveyed, including the entrance to each room in order to facilitate the posterior registration with the point clouds of the interior rooms obtained by the TLS. The photographs of the spiral stairs were processed using the photogrammetric multiview software Pix4DMapper [44] with automatic calibration of the camera. The use of Pix4DMapper instead of Agisoft Photoscan, which was used for the exterior walls as referred in 3.1.1, is related with the success of the bundle adjustment. With the same set of photographs, for the first section of the stairs, Pix4DMapper performed the triangulation successfully while Agisoft Photoscan was not able to achieve it. So, Pix4D Mapper was preferred to process the remaining sections.

**Figure 9:**
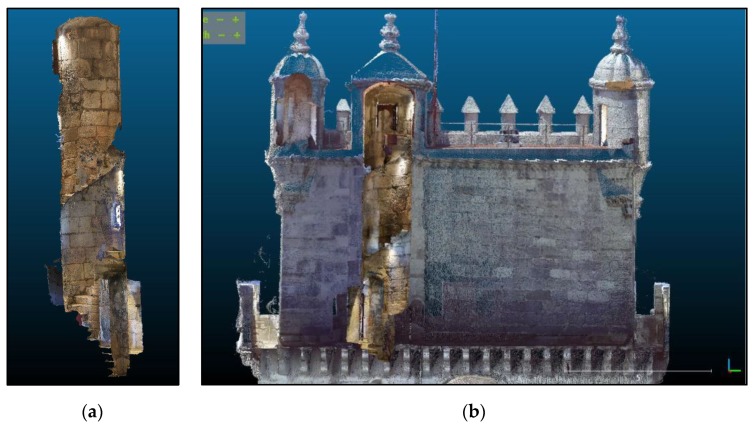
(**a**)Part of the point cloud of the spiral staircase between N05 and N06; (**b**) Point cloud of the spiral staircase aligned with main cloud.

A 3D point cloud of each of the four sections was obtained with an overlapping area with the next section (Figure 9a). In this way, partial clouds of the stairs could be registered to one another. None of the approaches used in the phase of image acquisition yielded a 100 % complete dense point cloud due to the complexity of the object, short distances and weak illumination conditions. The use of a flash did not improve the quality of the images because of reflections. Nevertheless, the continuous spiral sequence approach yielded a better result regarding completeness. The width of each door was measured “in situ” on the base of the arch in order to scale the cloud and facilitate the alignment with the main cloud obtained by TLS. The registration of the partial clouds and the final alignment were done using the software CloudCompare [42]. Several pairs of corresponding points (at least 3 well distributed) in each pair of clouds were interactively marked in common areas. For the alignment of stairs and main cloud, those points were situated around the doors and in the window sills which were located on opposite sides along the whole cylinder (doors in the east and windows in the west) (Figure 9b).

### 3.2. Terrestrial Laser Scanner

The methodology to apply depends on the size, distribution and geometry of the object to be surveyed [49,50]. For the Tower of Belém a Faro Focus 3D X330 terrestrial laser scanner [51] was used. This equipment has a maximum measurement speed of up to 1 million points per second and an accuracy of ±1 mm at a distance of less than 25 m. According to the technical specifications, its maximum precision is at distances of up to 150 m [51]. SCENE software from Faro [46] is used to register the scans. SCENE allows three registration methods, “Target Based” in which targets can be interactively measured or automatically detected, and they can be points and planes existing in the natural scene or artificial targets included in the scene during data acquisition (spheres or black and white boards). The other methods, “Top View Based” and “Cloud to Cloud” consist in an automatic registration between point clouds. In the present case the main method applied was the one using spherical targets to register scans. The registration based on correlation algorithms between two point clouds, achieves high-precision results, though the processing time for large tasks can be unbearable.

The degree of detail in which the documentation shall be made depends upon the nature of the object and of the end use of the data. With respect to the historical nature of the Tower of Belém, one of the purposes of the work is to analyse yet unidentified and undated construction discrepancies. At the same time, from the point of view of future maintenance plans there is a need for an adequate metric data source for a pathological study of the infrastructure. The level of precision required is relative to the specific case study and is a subjective assessment point of the technician who is going to develop the intervention project. In this case, in the absence of a technician’s directive, a relative register precision smaller than 3 cm, adequate for a factory building was also considered adequate for the present heritage building [52]. Of the stated objectives, the one that requires the greatest resolution of spatial data is that of dating the construction elements of the heritage object [52,53].

The configuration of the TLS survey consists in the establishment of the resolution and precision of acquisition and of the spacing between the scanner stations of the scans to be taken. In the present case, the scanner used has nine resolution levels described according to the dot space obtained for a dot mesh recorded at 10 m on a face perpendicular to the scanner. This configuration significantly influences the acquisition time. The acquisition resolution influences also the maximum distance at which the scanner can be distant from the target spheres so that they can be detected in the processing. This factor is directly conditioned from the size of the used spheres. For the present survey, 24 cm and 14.5 cm diameter spheres were used. The larger ones have been necessary to register scans that due to geometric constraints were more distant from the scanner. The resolutions that allow a higher geometric resolution in the documentation in a shorter scanning time are 1/5 and 1/4 (corresponding to 7.67 mm and 6.14 mm, respectively, at 10 m of distance). The precision parameter in the used scanner is an abstract term ranging from 1× to 8×, eventually related with the intensity of the laser ray (1× less intense to 8× more intense). We used 4× quality, a medium quality, which is recommended by the manufacturer for acquisition of scenes spaced from the scanner more than 20 m [51]. The scanning time for a full circle scan with 1/5 − 4× configuration with photo shooting is 8’34’’ and with the 1/4 − 4× configuration with photo shooting is 11’9’’. Higher resolutions or higher levels of precision raise the scanning time to 30’and lower resolutions or lower levels of precision make it difficult to detect the spheres during post-processing for the automatic registration. For those reasons, the 1/4 − 4× configuration with photographic acquisition was chosen to carry out the survey with TLS.

The scanning configuration determines the spacing between the scanner and the targets (spheres): in the case of 14.5 cm spheres it is 15 m and in the case of 24 cm spheres it is 28 m. If the objective of the geometric acquisition and the nature of the building are taken into account, the spacing between scanner positions is itself conditioned in order to obtain the complete geometry, aiming to obtain a cloud with the minimum of gaps. In this way, more 3D scans are needed than theoretically necessary. For the registration between scans there must be at least two common spheres and the scanner has to be level at the time of capture. The SCENE software performs the registration based on the correlation of distances between targets of individual scans, and also uses the record of the scanner’s clinometer as the “z” reference plane. The clinometer record is assigned a higher weight, in terms of quality, than that assigned to the reading of targets. In the survey carried out in this project, the number of spheres present at the scene was, in most cases, greater than 3.

The geometry and accessibility of the building conditions the planning of the survey. In the Torre de Belém, two groups of spaces are distinguished: in the first the execution of 3D scans can be consecutive and there is the technical possibility of registration from the reading of spheres (conditioning of the scanning maximum distance); in the second group that is not possible. It could be established that levels N-01, N00, N01, N02 and NC (Figure 6) could be scanned consecutively and registered by spheres. Levels N3, N4 and N5 could not be scanned consecutively, but they could be registered by reading targets (spheres or black and white boards) directly from level N01 (Figure 6). And the NE and N06 levels are independent, they cannot be linked through targets with the rest of the scan blocks. The described scan groups converge into three clusters. On the one hand, the register of levels N-01 to N05 and NC will be possible in a relative coordinate system, so that the georeferencing of this set is carried out for the whole block. On the other hand, there is the register of the scans taken in N06 and NE, which constitute two blocks of independent scans that will be registered to the rest from specific GNSS points determined for georeferencing. In total, 147 TLS scans were required for indoor and outdoor surveys, including those for georeferencing.

Three clusters were considered to generate the whole point cloud: a cluster comprising the scans that house levels N-01 to N05; a second cluster for the documentation of the upper terrace (N06); and a third cluster to house the scans of the Tower’s exteriors (NE) (Figure 6).

Table 1 shows the list of registered scans, distance between scanner positions and scanning configuration for the different levels structured according to Figure 6. The distance between scanner positions for the execution of the survey is the result of the need to obtain a determined level of detail. The resolution/quality setting only varies for the scans taken at level N-01. This is because this level is a warehouse that was occupied by furniture and stored objects. In the interior room levels, scanning at the highest resolution is chosen in order to obtain a survey in as much detail as possible.

The distance between scanner positions is short because it is intended to cover as many areas as possible avoiding gaps. At certain levels there are rooms and surveillance spaces that have to be individually scanned (N01, N02, N03, N06). Therefore, the number of scans to be performed at these levels is increased. In N05 there is a perimeter balcony that increases the number of scans. While for interior scans the scanner configuration 1/4 − 4X was used in order to obtain very dense point clouds, on the exterior levels, the same scanning configuration has been chosen by necessity, given the height of the tower (≈30 m) and the distances between scanner and object. By scanning the upper areas of façaades, the reading angle is not the most suitable and the distance to the façade is greater than in the interior settings eventually leading to loss of resolution if this is not compensated by the scanner configuration.

#### 3.2.1. Interior Rooms

**Figure 10:**
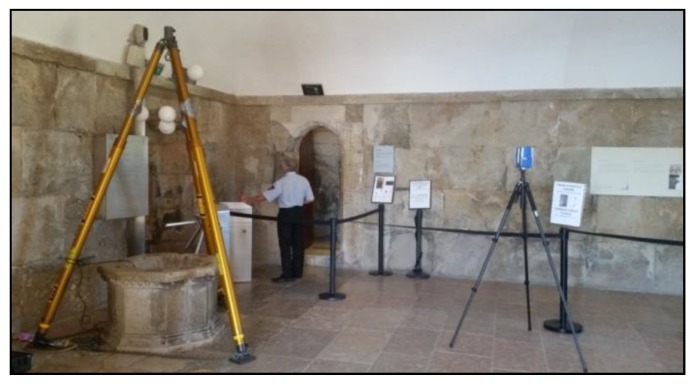
Structure required to access the cistern.

The tower has seven levels accessible by stairs and one level (cistern) accessible by other means (Figure 10). To register different spaces with a TLS there must be enough space to make scans in which the field of view is broad enough to capture part of the two spaces to be registered. As already mentioned, the connection of levels N02 to N05 is by means of a spiral staircase, the dimensions of which make it impossible to use the laser scanning technique to register the different levels. This made it necessary to seek support in the first terrace (N01), that can be sighted from the levels above, to register the clouds that refer to the rooms at levels N02 to N05. The support in the first terrace was made through spheres of 24 cm of diameter in the central area. These spheres were viewed through balconies and windows at the different levels, so at each level a registering scan had to be made near the window or balcony simultaneously capturing a part of the interior of the room (with two spheres) on one side and the terrace N01 with the reference spheres on the other side.

When a high-resolution survey is performed, scans are acquired at station distances not greater than 10 m from one another, thus aiming to capture the maximum detail of vaults and reduce hidden areas. Level N00 (entry level) is made up of multiple vaults delimited by elliptical arches. For maximum detail a scan was needed for every pair of vaults, which resulted in 23 scans at this level. The only areas that remained hidden were behind the existing cannons because these were too heavy to move.

Level N00 includes a small inner courtyard, previously accessible through open arches all around, but which is now closed by terrace doors. For the documentation of the inner yard 6 scans were made to fully cover the space. The register to the interior of the level was made through different doors, which were opened for the scans to be made. Level N-01 is below the water line and can be accessed by the service stairs. This level includes three low-ceilinged rooms, two on the east and west sides and one central room. For the full documentation 25 scans were required at this level due to the narrowness of the passages.

Except for the six watchtowers, the first terrace (level N01) was defined in four scans. For the watchtowers, individual scans were made inside each. As already described, in this first terrace a GNSS station and six control points were set up, requiring five more scans to georeference the point clouds. From the control points located at the N01 level, the cluster containing levels N-01 to N05 (interior rooms) was georeferenced. The interior of levels N02 to N05 is similar, a square space with small divergences (terraces, watchtowers or balconies). At the N02 level, six scans were made, eight at N03, seven at N04 and 10 at N05, which included its surrounding balcony.

The cistern (NC) is located under the floor of the N02 level and it can only be accessed through the mouth of the well, which serves to monitor the water level and for drawing water. The Lisbon fire brigade collaborated by verifying air safety in the normally closed space and helping to access it. Access was gained by descending with the equipment down to the ground using a tripod with pulley placed over the mouth of the well. The registration of the scans made inside the cistern and at level N02 was assured by scanning several spheres installed in the mouth of the well visible from below and later scanning the same spheres from level N02. In order to achieve a precision registration between the two levels the tripod had to be very stable and the maneuver of the firefighters agile when lowering and raising the operator of the laser scanner and the equipment itself. The operation consisted of scanning the interior of the NC level with two scans, one just under the entrance to the cistern (the well) and a second one in the opposing position in the room. Finally, equipment and operator were removed, taking care not to move the spheres installed at the well mouth, and a new scan was made for the registration with level N02.

#### 3.2.2. Exterior Walls and Top Terrace

The documentation of the upper terrace (N06) and the exterior of the tower (NE) facing land was made independently of the interior of the tower. Access to the upper terrace is provided through the narrow spiral staircase which makes it difficult to register the point clouds by spheres. Clouds could not be registered through the spheres used in the N01 level either, since such a distance required scans of long duration (higher resolution to detect the spheres) or the use of larger spheres, which were not available. The survey of this upper level requires a greater number of scans given the existence of four watchtowers that have to be expressly scanned from the inside to carry out a complete documentation. In addition, it is required to obtain extra scans for reading control and check points for georeferencing level N06. Four scans were needed for the terrace (N06), and a further four, one inside each of the existing watchtowers. The level was georeferenced by five control points determined by GNSS. The idea of registering NE to the remainder over the existing access bridge to the N00 level was also considered (Figure 6). This option was dismissed due to the weak stability of the platform in relation to the needs of the scanner during operation. The exteriors were completed with 12 scans from land, which included six control points along the perimeter of the tower. The survey was carried out from scanner stations separated by less than 25 m. Spheres of 24 cm and 14.5 cm were used to ensure a solid union allowing a scan to be registered to the following two and to the previous two, whenever existing.

### 3.3. Georeferencing Photogrammetric and TLS Clouds 

**Figure 11:**
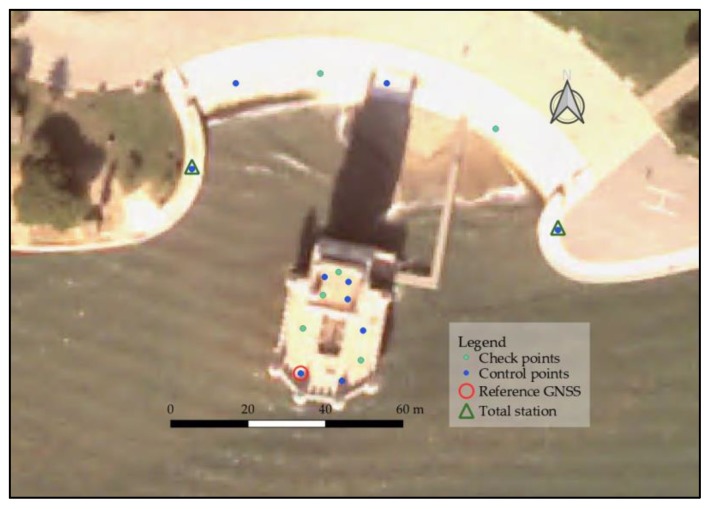
Location of control and check points.

The relative positioning of the point clouds is achieved through the georeferencing of the point clouds obtained individually by each method. For this, a point network was established with GNSS measurements longer than 15 min in static positioning. The acquired support for the georeferencing was adjusted to the space available for determining GNSS points and to locations where there were no multipath effects or other possible problems presented by the GNSS technology. The points arranged for georeferencing the group N-01 to N05 were located in N01. In N06 the available area was small (~10 m ×10 m), so the distribution of control and check points was not ideal. In NE the points were distributed along the outer perimeter of the walkway surrounding the tower on the north side (Figure 11).

The network points were materialized on the exterior of the tower by means of nails in the ground, and with marks made on the floor in the terraces of the Tower (which were erased once the work was completed). In order to obtain a greater precision in the determination of the network points, forced centering was used, thus reducing eventual miscentring. To calculate the points, a reference was established in N01 (Figure 6 and Figure 11) with a static post-processing GNSS positioning longer than 4 h, calculating its position with respect to the network of permanent GNSS stations (RENEP) of the Direção Geral do Território (DGT). For the rest of network points, an intersection was made between the baselines from the reference, the nearest RENEP station and the GNSS observed points. The coordinates obtained for all points were determined in the official coordinate reference system of Portugal, ETRS89 PT-TM06, with orthometric heights. 

The scanner generates scaled point clouds, that is, when a fixed block containing “n” scans is georeferenced, they are locked in relative position and a rotation and displacement is applied to all of them. Unlike TLS, the photogrammetry, when there are no control points considered in the bundle adjustment, generates point clouds in a local and arbitrary coordinate system, and the generated cloud is not scaled. This implies that in the georeferencing process there is a displacement, rotation and scale of the point cloud to be determined. Table 2 shows the distribution of control and check points used for the georeferencing of the different point cloud groups.

For the point clouds originating from photogrammetry, several approaches were tested conditioned by the fact that it was not possible to place marks in the exterior walls facing water, because of its inaccessibility, nor in the spiral stairs because of monument management restrictions. For the bulwark exterior walls, a set of 16 conspicuous points (control and check points) was used, eight in the east and eight in the west façade.

In the first approach, the scaling and alignment of the cloud was done in CloudCompare [42] using the georeferenced TLS point cloud as a reference. After an approximate alignment by common point pairs in the east and west façades, an IPC adjustment of the photogrammetry cloud to the reference cloud was performed. In the second approach, the usual way in photogrammetry was followed: the coordinates of all 16 points were determined with a total station from the areas closest to the walls situated on the promenade (Figure 11) and input in the bundle adjustment. A third approach consisted in picking up the coordinates of the same points from the georeferenced TLS point cloud and introduce them in the bundle adjustment. The first and third approaches were a test to the possibility of dispensing the measuring of control and check points in the field. The software used for the second and third approaches was Agisoft Photoscan [43].

To georeference the point cloud of the spiral staircase, the first approach was used, alignment through identic point pairs, as already mentioned in Section 3.1.2, but without using the ICP algorithm since there were only small common areas between the stairs and the main georeferenced TLS point cloud. The manually measured point pairs were well distributed along the staircase cylinder, in five windows and five doors.

### 3.4. Hyperspectral Camera and Spectroradiometer

The spectral data was acquired in a two-step process: first, hyperspectral tools were used to determine the areas of interest, and then a spectroradiometer was applied to obtain a precise characterization of the materials forming the analysed object. The hyperspectral images were exclusively taken to ensure that the rocks where data will be taken from with the spectroradiometer correspond to the material to be studied. Organoleptically, its similarity can be appreciated, but by making a classification using the Multi-Layer Perceptron (MLP) backpropagation neural network, providing endmembers taken on the image itself, we assure that the visual similarity corresponds to the spectral reality.

In order to find the possible origin of the ashlars used in the Tower of Belém, several scans were made of the exterior walls of the monument using a Xenics Xeva-2164 hyperspectral camera, a Specim Imspector N17E image spectrograph and a Xenics Infrared Solutions mirror scanner reading system. The same instruments were used for the measurements in the quarries. The hyperspectral camera is characterized by having a passive sensor that captures the electromagnetic energy reflected from the surface of the object and by having a scanning sensor consisting of the oscillation of a mirror [54]. The spectral range of the camera is from 900 nm to 1700 nm. The CCD sensor is 14.2 mm × 4.4 µm in size and has a spectral resolution of 5 nm. The sensor collects information from a scene on the axis perpendicular to the rotation of the mirror, thus minimizing any directional effects on the images [55]. The camera was positioned at a distance of 9 m from the quarry fronts. The light source was the sun and all surveys were performed within a short timeframe in order to obtain the images under similar light conditions [56].

The data cube of the hyperspectral camera has 320 pixels per row, “n” pixels per column (the number varies depending on the range of the angle of rotation of the camera mirror) and 256 bands. This cube was normalized in IDL software and undergoes a learning training through neural networks with a MLP supervised model in the software ENVI [57]. It has been trained with a backpropagation algorithm. An MLP network is a structured artificial neural network with an input layer, one or more hidden layers, and an output layer. These layers are connected to each other by a series of weights, thus establishing the relationship between them. The information obtained from the input layer is processed by the hidden layers and sent to the output layer. MLP is a non-parametric, non-linear regression statistical model [58].

The network architecture used is configured with an input layer containing 256 units, one per band. Two hidden layers, with processing units, are connected to the input layer units and the next hidden layer. This second hidden layer receives the information from the first layer, processes it and sends it to the output layer. The output layer contains the classes with which the image will be classified (reference white, reference black and Lioz from the Tower of Belém).

Following this process, a classification is made of the different geological materials appearing at the quarry fronts. A “Region of Interest” (ROI) is generated, which contains as many classes as there are separate materials appearing at the scene, including reference black and white. Each class is provided with the necessary information for the learning process and in order to do so pure pixel data of each class (endmembers) were collected beforehand.

The used backpropagation training method compares the output signal with the expected value and calculates the error, which is fed back by the neural network to modify the weights of the connections and thus be able to minimize said error [59]. This process is repeated until the neural network achieves a weight adjustment whose error parameter is tolerable [60].

The neural network has been used with the ENVI software and configured with a "Logistic" activation function. Training threshold contribution determines the contribution size of the weight relative to the activation level. This has been set to 0.9. The configured training rate field is 0.05, a low value to avoid assuming the risk of oscillations and the non-convergence of the result in the training process. Training momentum, set to 0.8, establishes a higher rate and without oscillations, with higher steps. The RMS Exit Criteria is established as 0.0001, so that the training process stops when it reaches this value. This RMS Exit Criteria has been established to increase the required level since other studies cite values of 0.05 [61]. Minimum output activation is 0.6. If the classified pixel has a lower value it will be labeled as unclassified pixel.

Data from the limestone were then captured using the spectroradiometer ASD FieldSpec4 to obtain the reflectance curves. This equipment has a spectral range between 350 nm and 2500 nm. Its spectral resolution is 3 nm for the range VNIR (350–1000 nm) and 10 nm for the range SWIR (1001–2500 nm) with an accuracy of 0.5 nm [62]. Local conditions as well as meteorological conditions for the spectroradiometer were the same as for the hyperspectral camera.

The areas where the Lioz spectral data have been taken have been selected for meeting the optimal environmental lighting requirements for valid capture. Previously, the entire monument has been organoleptically inspected, observing that the material used for its construction was very homogeneous. Based on the classifications obtained from the hyperspectral data and the neural network, four experiments were designed: Tower of Belém, Rio Seco quarry, Alvito quarry and Aqueduto das Águas Livres quarry. Before each experiment the equipment was environmentally stabilized for 20 min and the sensor optimized using the reference white (spectralon), assigning to it the maximum reflectance value (99.99%). Taking into consideration earlier work with this radiometric equipment [63] as well as its technical characteristics, an optimum distance of 4 cm from the wall or quarry front was established, which provided an effective sampling area of 3.2 cm^2^.

**Figure 12:**
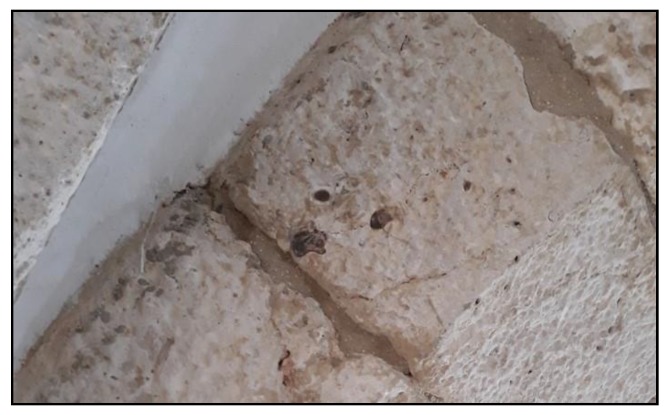
Silica (dark spots) found in an ashlar of the Tower of Belém.

At each of the four sites a series of shots were taken: 120 in the Tower of Belém, 95 at the Rio Seco quarry, 35 at the Alvito quarry and 35 at the Aqueduto das Águas Livres quarry. The mean value of the data of each different material was calculated for each site. A Gaussian smoothing was then applied to eliminate noise and to spectrally define each material using the software UnScrambler [64]. Lastly, as various nodules of hydrated silica were found in some ashlars, a series of radiometric shots was taken in order to compare them with the ones measured at the Rio Seco quarry (Figure 12).

## 4. Results

### 4.1. Geometric Study

The complete 3D documentation of the building was obtained from the surveys including all the rooms and cistern. As a result of the registration of independent clouds, the geometry of the Tower of Belém was collected in the form of approximately 6200 million points. As for the photogrammetric surveys, the south wall was included in a cloud of 8.5 million points and coverage of the spiral stairs produced 1.5 million points.

**Figure 13:**
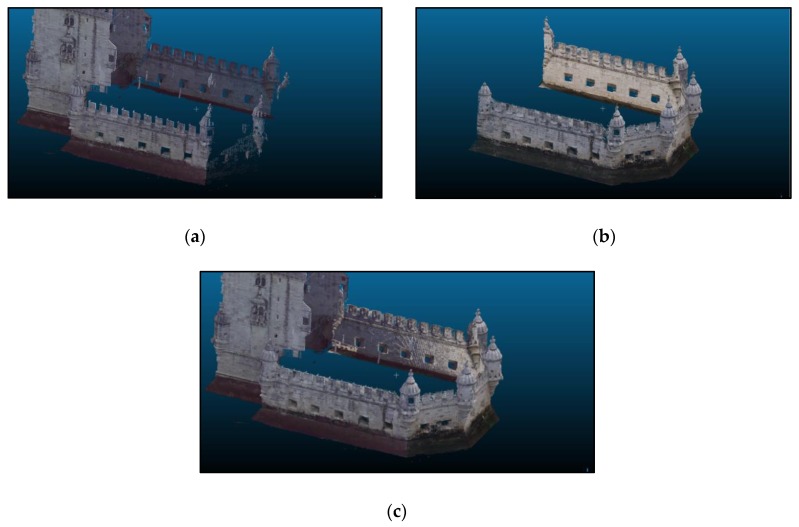
Point clouds from different sources: (**a**) from TLS; (**b**) from close-range photogrammetry; (**c**) same clouds after georeferencing of the photogrammetric point cloud. The upper left corner is pointing North.

**Figure 14:**
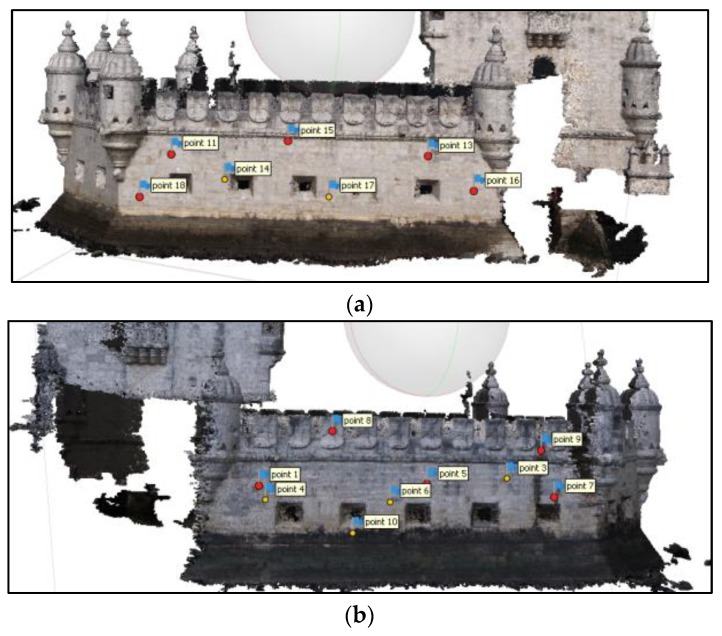
Silica distribution of control (red) and check (yellow) points for georreferencing the photogrammetric point cloud. (**a**) East side; (**b**) West side.

**Figure 15:**
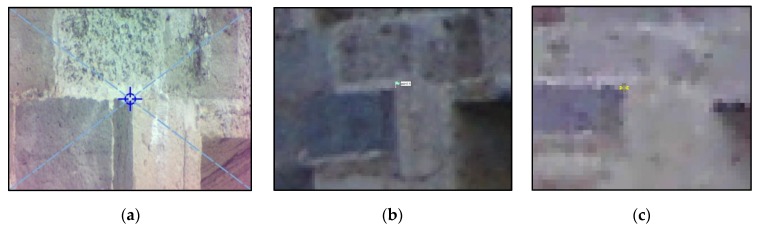
The same control point as appearing: (**a**) the total station image; (**b**) one photogrammetric image; (**c**) the TLS point cloud.

Figure 13 shows part of the georeferenced TLS point cloud (a), the photogrammetric point cloud (b) and the resulting cloud (c) after georeferencing the latter. As one can see, the overlapping area is significant consisting in the whole east and west sides of the bulwark. The objective of the photogrammetric survey was to add the south side to the main 3D point cloud completing the model. East and west sides were used for locating control and check points and for cloud to cloud alignment in the approaches followed for georeferencing, mentioned in Section 3.3. Figure 14a and Figure 14b show the distribution of control and check points. An example of control point identification in the image taken by the total station, in one of the photogrammetric images, and in the TLS point cloud is shown in Figure 15.

Table 3 shows the quality of the achieved absolute orientation evaluated by the RMS values at control and check points, where CA means Cloud-to-cloud Alignment and BA means Bundle Adjustment.

**Figure 16:**
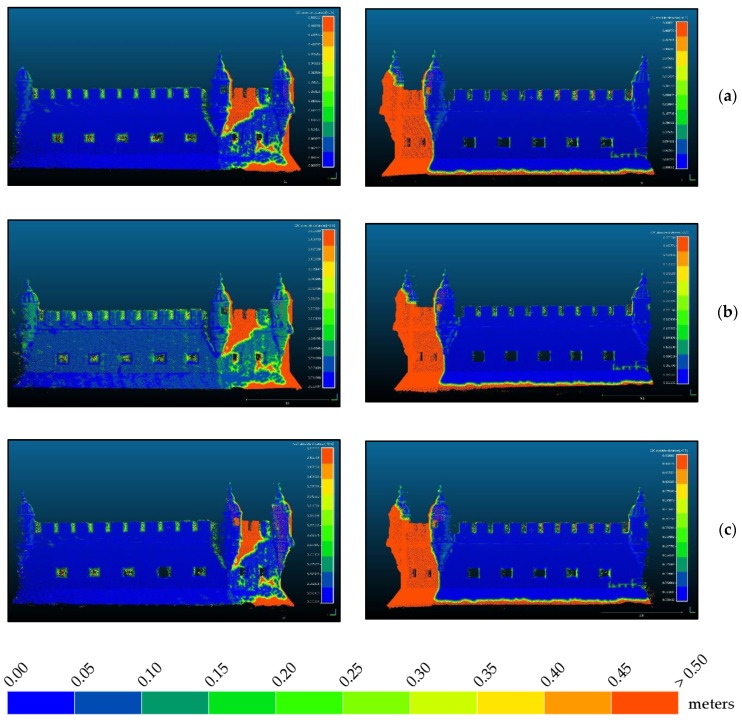
Differences between photogrammetric and TLS point clouds in common areas: (left) West bulwark façade; (right) East bulwark façade. (**a**) ICP; (**b**) Total station points; (**c**) TLS points. (Bottom) enlarged color scale.

**Figure 17:**
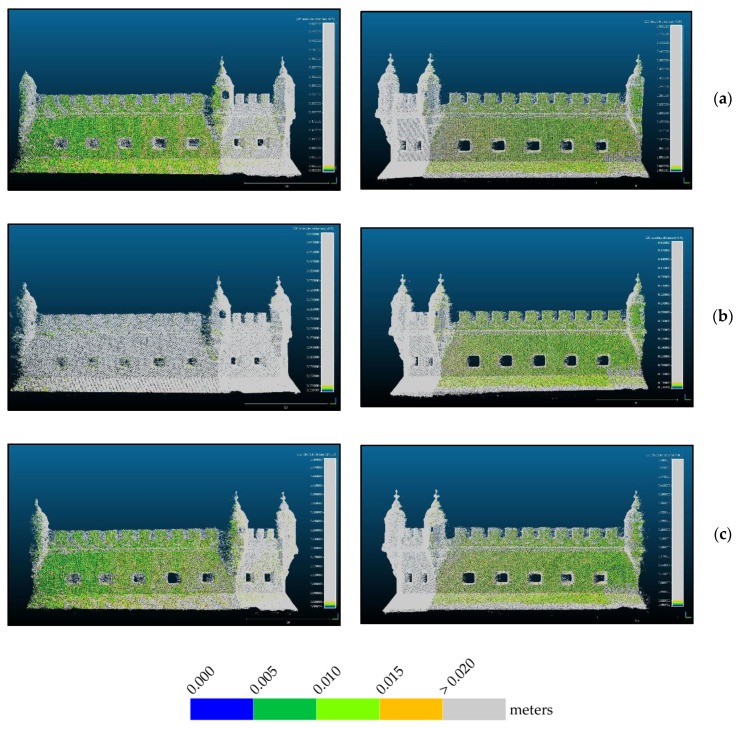
Distribution of points where residuals are smaller than 2 cm: (left) West bulwark façade; (right) East bulwark façade. (**a**) ICP; (**b**) Total station points; (**c**) TLS points.

**Figure 18:**
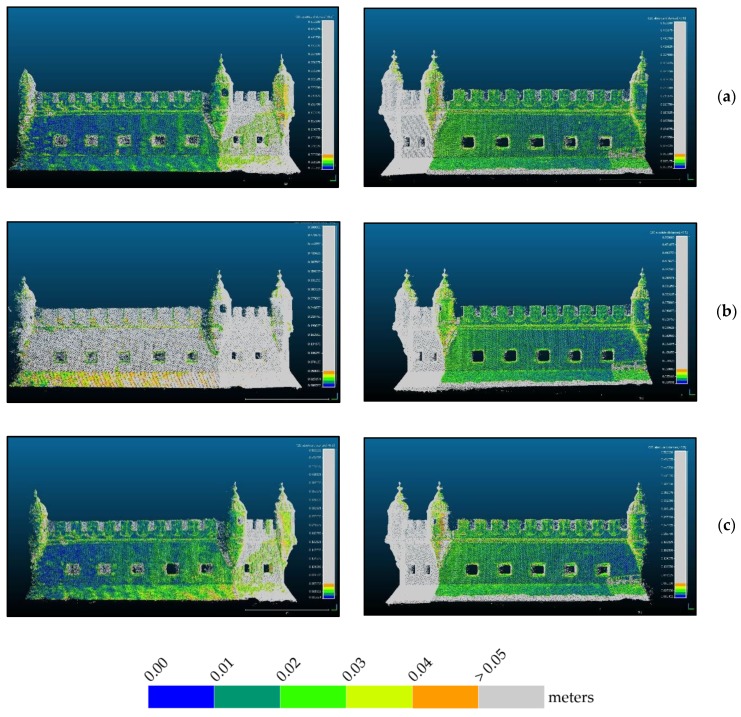
Distribution of points where residuals are smaller than 5 cm: (left) West bulwark façade; (right) East bulwark façade. (**a**) ICP; (**b**) Total station points; (**c**) TLS points.

Due to the problems associated with the registration of point clouds originating from different techniques, further than trusting only in RMS values calculated on 16 points, the quality of the three adjustments was analyzed for the whole common areas of photogrammetric and TLS point clouds. Figure 16 to 18 show the differences between the two clouds, TLS and photogrammetric, after georeferencing the latter with each of the three approaches.

From the analysis of Figure 16, it is evident that most regions are blue or dark green denoting most differences are smaller than 10 cm. Regions in red are not to consider since they correspond to points that are not existing in the reference TLS cloud. The east façade, (right column), shows smaller differences in all approaches than the west façade (left column), thus appearing in a darker blue. The thin red stripe on the base of the bulwark that appears in all images in the right column is due to the presence of water, showing that the tide during the photogrammetric survey was lower than during the TLS survey of the east side. Water absorbs infrared laser so that there are no surveyed points in water. On the left column there is no red stripe on the base, indicating the tide during the TLS survey of this side (not simultaneous to the other) was similar to the one existing during the photogrammetric survey. Obvious is also that the approach shown in (b), georeferencing with control points measured with a total station, does not behave so good as the other two (mostly green instead of blue) although it presents the smallest RMS at control points (Table 3). In order to increase the resolution of the color scale, Figure 17 and Figure 18 present only the map of points with differences to the reference smaller than 2 cm and 5 cm respectively.

From Figure 17 we see that in: (a) there is a homogeneous distribution of the points with residuals smaller than 2 cm, being the most even smaller than 1 cm. In (b), especially in the west façade (left column) almost all points present residuals greater than 2 cm. Approach (c) presents an inhomogeneous distribution of residuals both spatially and in magnitude. The fact that the west façade behaves worse than the east façade can be due to the lighting conditions during survey. Figure 14 shows the difference in lighting existing between east (Figure 14a) and west (Figure 14b) façades. Since the camera had to compensate for the less light in the west side increasing exposure, it is possible that, being on a vessel, the waves movement during a longer exposure affected the image sharpness. Homologous points detection algorithms are very sensitive to blur and some noise appearing in the point cloud can be originated by blur caused by the acquisition conditions, explaining this result. 

Figure 18 corroborates the analysis done for Figure 17. In the (b) approach, west façade, differences are even bigger than 5 cm, while the other maintain their behavior, letting us conclude that the best adjustment is the one illustrated in (a), which corresponds to the ICP algorithm for cloud alignment, performed with Cloud Compare. This way we consider the RMS value of this transformation, 2.72 cm, as the accuracy of the georeferencing of the photogrammetric point cloud.

An estimate of the overall error based on the different methodologies used is shown in Table 4 and Table 5 compiles the errors obtained in the georeferencing of TLS surveys in control and check points. The union of all laser scans georeferenced through points with GNSS coordinates presents a maximum error of 1.2 cm.

**Figure 19:**
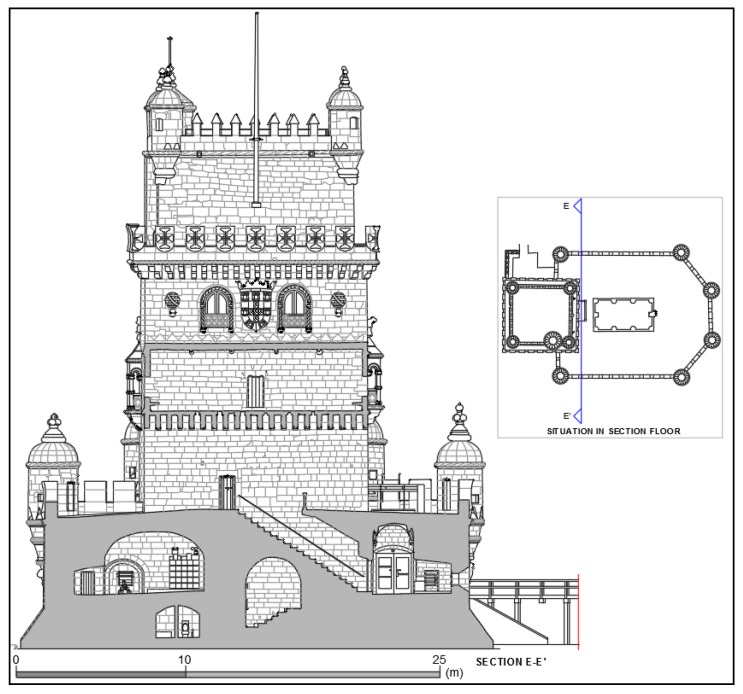
Elevation drawn from a vertical slice tangent to the Gothic tower south facade.

The complete geometry of the tower is the source from which detailed plans and elevations obtained from cloud cuts can be generated in any desired direction. Given the spatial resolution of the documentation, the products generated reveal details of elements such as vaults and floors. Figure 19 is a projection of the tower on a vertical plane viewed from the south, where both photogrammetry and TLS were integrated.

### 4.2. Spectral Analysis

**Figure 20:**
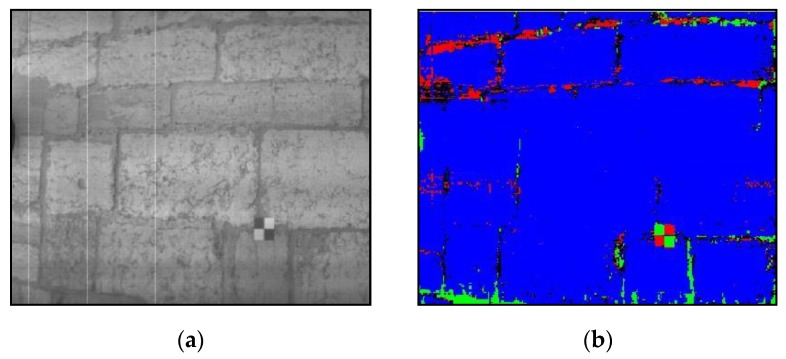
(**a**) Hyperspectral image; (**b**) Material classification obtained with a neural network (Blue = white Lioz limestone).

The data obtained from the quarries and the Tower of Belém using the hyperspectral camera (Figure 20a) underwent a classification process by means of an MLP neural network. It was thus ensured that data collection by the spectroradiometer would be carried out in the areas where the study object was located (Figure 20b).

**Figure 21:**
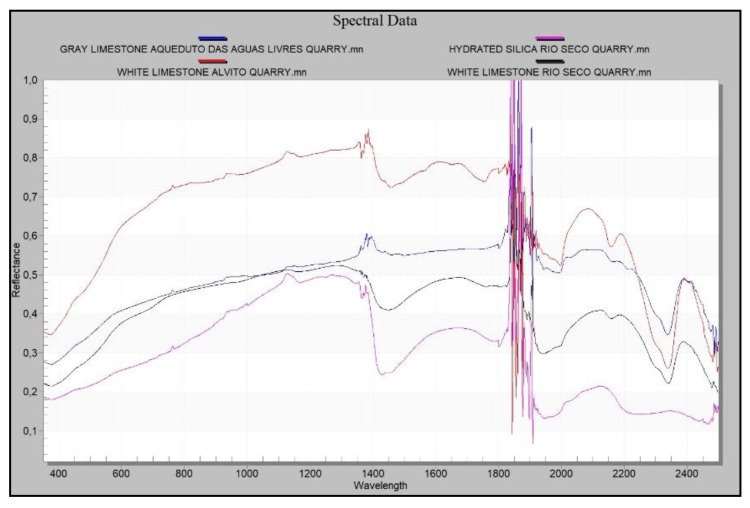
Reflectance spectral signatures of materials in the quarries. Gray limestone from Aqueduto das Aguas Livres Quarry (Blue) and hydrated silica Rio Seco Quarry (Purple) differing significantly from the white limestones in Alvito Quarry (Red) and Rio Seco Quarry (Black).

Spectral data are acquired using the spectroradiometer because its spectral range is larger and its accuracy better than those of the hyperspectral camera. The spectral curves of the materials of the quarries were compared (Figure 21). From the classification process by the described method, a RMS Exit Criteria of 0.00184 is obtained. This value is very low and indicates that the root mean square error obtained in the process is acceptable. The classified image contains 115,520 total pixels of which 92.151% of pixels have been classified correctly. Therefore, the purpose of this process is to determine the homogeneity of the analysed material, in this case it is confirmed that the analysed scene is Lioz.

**Figure 22:**
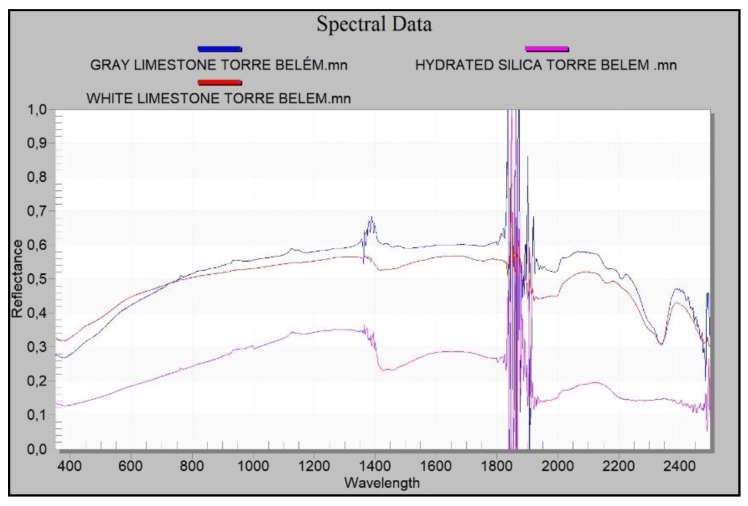
Reflectance spectral signatures of ashlars in the Tower of Belém.

The materials measured at the Tower of Belém present spectral similarities with the exception of hydrated silica, whose signature differs from the carbonates (limestone) since it is a silicon oxide. Silica shows lower reflectance and its absorbance peaks do not correspond to those in the carbonates in the limestones (Figure 22).

### 4.3. Damage Study

In 1997 the Tower of Belém was rehabilitated and consolidated [65]. This intervention focused on solving some structural problems (misaligned elements and seriously fractured and deteriorated ashlars) as well as on cleaning and improving the construction materials throughout the outer walls of the Tower (problems deriving from water leaks on horizontal surfaces, dirt from air contamination and biological colonization), and to repair deteriorated ashlars.

**Figure 23:**
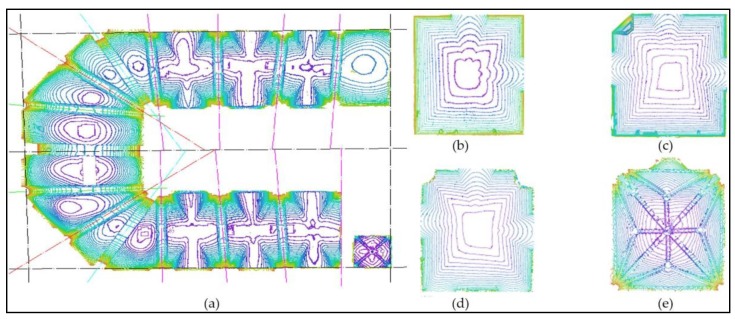
Height contour geometry of the vaults in the Tower of Belém: (**a**) Bulwark vaults–Level N00; (**b**) Level N02; (**c**) Level N03; (**d**) Level N04; (**e**) Level N05.

The present geometric evaluation reveals that the geometry of the structural elements (walls and vaults) is correct. Figure 23 shows a representation of the vaults in their present state in the form of height contours. The vaults of the bulwark (Figure 23a) present stable geometry without significant deformations, although there is an evident misalignment of the vaults when seen as a plan view. This misalignment indicates staking errors during construction at the base of its geometry and not the existence of structural problems. Regarding the vaults in the rooms of the tower (Figure 23b–e), their geometry is regular, with few deformations and without evident structural deficiencies.

In a visual inspection of the monument 20 years after the significant intervention, slight damages to the materials are visible, such as loss of material in construction elements, surface dirt produced by the deposit of particles on walls both outside and inside the tower, injuries from biotic attacks, mainly on the outside of the tower in the part that is flooded or near the water, and water leaks inside the tower.

## 5. Discussion

The geomatic survey of the Tower of Belém provided a 3D point cloud model for the execution of a geometric study of the entire monument. The maximal error in the point cloud would be ±2.7 cm. A spatial point cloud resolution of<1.5 cm outdoors and<0.6 cm indoors has been obtained. The constructive framing of the studied monument is unique as it required the development of an appropriate method for the implementation of techniques and equipment in the geometric survey. The combination of close-range photogrammetry and TLS leads to the recognition of the advantages of TLS for surveying interior spaces, and of the close-range photogrammetry, in this case, carried out from a vessel for the areas of the monument difficult to access or not visible from any land point to be surveyed by TLS. The method used, assuming a combination of techniques of different nature, does not allow establishing an overall value of accuracy for the entire 3D point cloud. The accuracy obtained must be individually considered depending on the technique used, and in the case of photogrammetry, depending on each project (exterior facades and spiral staircase). Nevertheless, knowing that 80% of the work is done with TLS, 12% with close-range photogrammetry for the facade facing the river, and 8% with close-range photogrammetry for the spiral staircase, the global error of the TLS can be considered the most representative for a global accuracy of the 3D representation, so that a weighted average error of ±1.38 cm can be assumed for the project. 

Regarding the georeferencing errors obtained (Figure 4 and Table 5), the higher error obtained in the close-range photogrammetry project for the exterior façades of the tower must be highlighted. This error is determined by the light conditions given at the time of data collection. In Figure 14, it can be seen that one of the facades is less illuminated than the other, which forces to use a longer exposure time in the photographic capture, causing the images to be more affected by the movements of the ship reducing its sharpness. Despite masking all the images to avoid correlation errors at far points (sky and water), and eliminating all the images that were found with significant blur, the absolute precision could not be reduced to better values. Noteworthy is the contradiction between RMS values on control points (Table 3) and residuals between georeferenced point clouds. Figure 16 to Figure 18 show that in spite of yielding a better RMS, the bundle adjustment with control points measured with a total station behaves worse with respect to residuals than the bundle adjustment with control points retrieved from the TLS cloud.

As for the spiral stairs, the use of marks on the walls that could be determined by sightings through the door openings could have improved the georeferencing results. The use of marks was not possible since, due to maintenance works, the photographic survey of the stairs was made in several epochs along one year and it was not allowed to leave marks in the monument. Nevertheless, the achieved accuracy upon alignment (±1.22 cm) is comparable to the absolute accuracy of the main point cloud. This result could not be checked with control points.

The 3D point cloud model obtained has a mean resolution of≈1 cm. This made it possible to document the status of conservation of the Tower of Belém almost entirely as well as the ashlars that make up the structure and walls. This documentation sets a reference regarding the state of conservation of this heritage building, since it includes both interior and exterior of the tower. Any intervention or subsequent study may be compared with it and the degree of deterioration suffered over time can be identified and quantified.

Upon inspection, damage deriving from the visual alteration of constructive elements (humidity, surface dirt by surface washing and deposit or biotic attacks resulting from the action of the ocean) could be seen, as well as some erosion damage as a result of use or weathering. The nature of the damages observed is similar to those surveyed prior to the intervention carried out in 1992, revealing that the action of the ocean and meteorological effects, as well as the location of the monument, determine the appearance of these features.

The geometric study facilitated the assessment of the structural state of the tower. The structural system is made up of load-bearing walls and vaults. Thicknesses of walls, floor geometry and elevations as well as the geometry of vault surfaces were determined. The geometric study of the vaults reveals the underlying structural health of the building, as reflected in the absence of significant deformations or constructive deviations with respect to its theoretical geometry.

**Figure 24:**
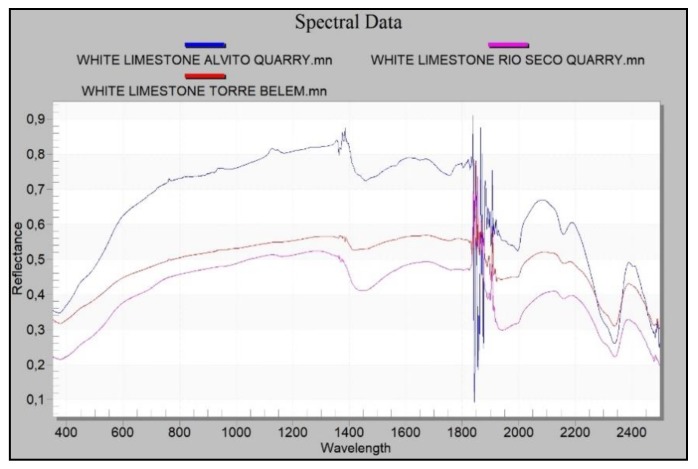
Spectral comparison between Lioz white limestone in the quarries and in the Tower of Belém. Evidence of spectral similarity of absorption peaks and slopes between the limestone at Tower of Belém (Red) and the limestone in Rio Seco Quarry (Purple).

As for the results obtained from the spectral signatures of the materials in the Tower of Belém and in the quarries analysed (Rio Seco, Alvito and Aqueduto das Águas Livres), the results of the reflectance and absorbance of the materials making up the white limestone ashlars in the Tower of Belém matches with the materials of the Rio Seco quarry. There is also spectral similarity between a silica nodule in one ashlar and the silica found at the same quarry. The same cannot be said of the results of the Alvito quarry. The reflectance is similar to the other white limestones, but the signature shows different slopes. This fact means that there is less likelihood that the material to build the Tower of Belém was extracted from the Alvito quarry. The carbonate from this latter quarry is similar to the others analysed in this study, but it shows differences that prevent corroboration with the hypothesis that it was used in the construction of the Tower. The study lends more weight to the hypothesis that the material of the Tower of Belém came from the Rio Seco quarry, since the spectral signature is similar to that found in the Lioz used in the Tower of Belém (Figure 24).

**Figure 25:**
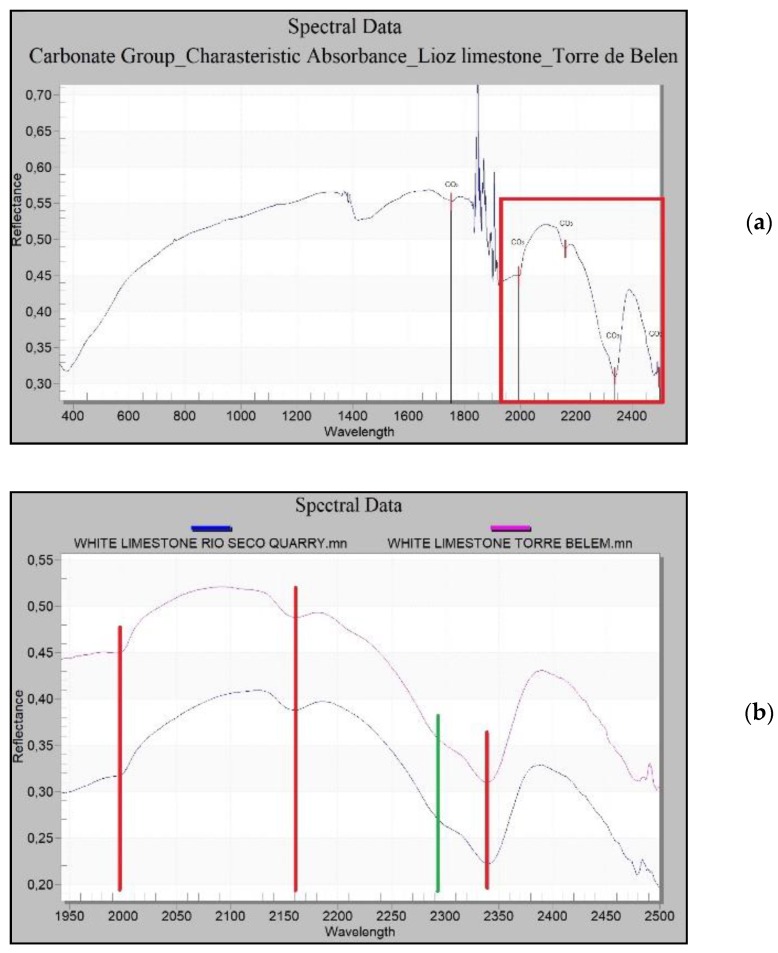
Comparison between the samples from the Tower of Belem and from the area of the white limestone in Rio Seco quarry. (**a**) Absorbance peaks of the carbonates group (CO_3_) of the white limestone of the Tower of Belém; (**b**) Enlargement between wavelengths 1900–2500 nm for comparison. Common absorbance peaks (Red) and slope change (Green) for Tower of Belém and Rio Seco quarry signatures.

Owing to the nature of these limestones, their characteristic absorption peaks appear at 1750, 1980, 2160, 2340 and 2500 nm [66] (Figure 25a). By analysing with increased resolution the range presenting more information about carbonates, which is between wavelengths 1900 nm and 2500 nm, the absorbance peaks of the Lioz white limestone at the Rio Seco quarry and in the Tower of Belém lie within the same wavelengths and correspond to those previously mentioned, which are characteristic of the group of carbonates. 

Common to the carbonates of both the Tower of Belém and the Rio Seco quarry, there is a change in the slope of the curve at 2292 nm. This reinforces the case for this quarry as the source of the Lioz supply for the construction of the Tower of Belém (Figure 25b).

**Figure 26:**
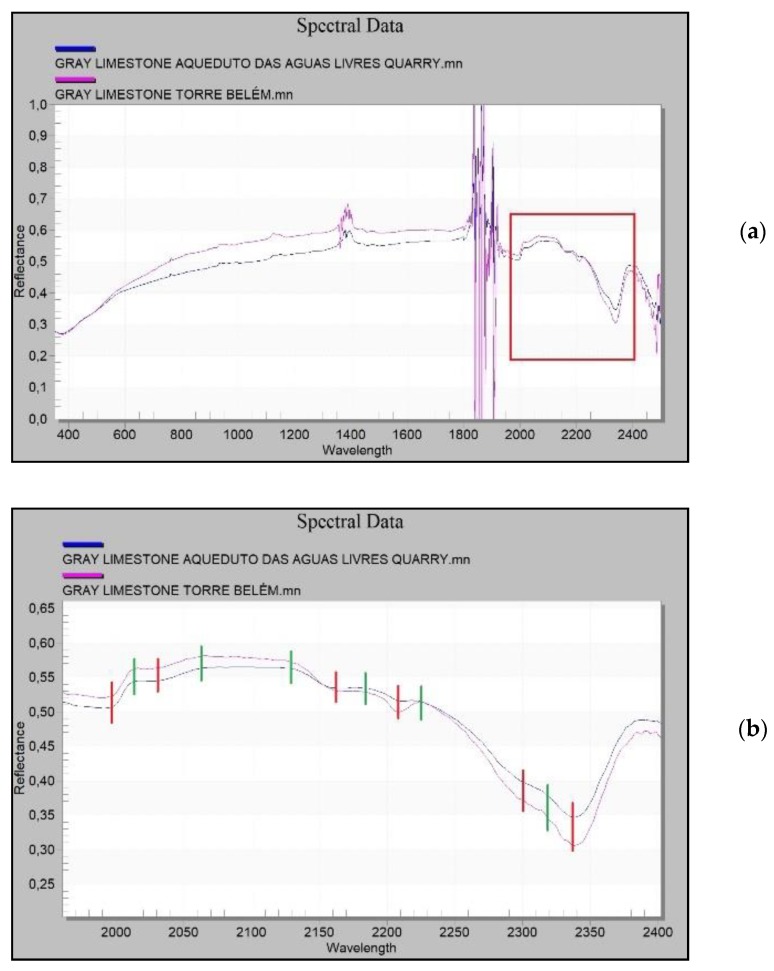
Comparison between the samples from the Tower of Belem and from the area of the gray limestone in the Aqueduto das Águas Livres quarry. (**a**) Similarity between spectral signatures of the gray limestones; (**b**) Absorbance (Red) and reflectance (Green) peaks for the gray limestones studied between 2000 and 2400 nm.

As for the gray limestones found in the Tower of Belém, these show reflectance values that are very similar to those found at the Aqueduto das Águas Livres quarry (Figure 26a). Absorbance and reflectance peaks of the gray limestones in the Tower of Belém and the quarry are found at the same wavelengths. These reflectance values confirm it is a carbonate and that the geological material is the same (Figure 26b).

**Figure 27:**
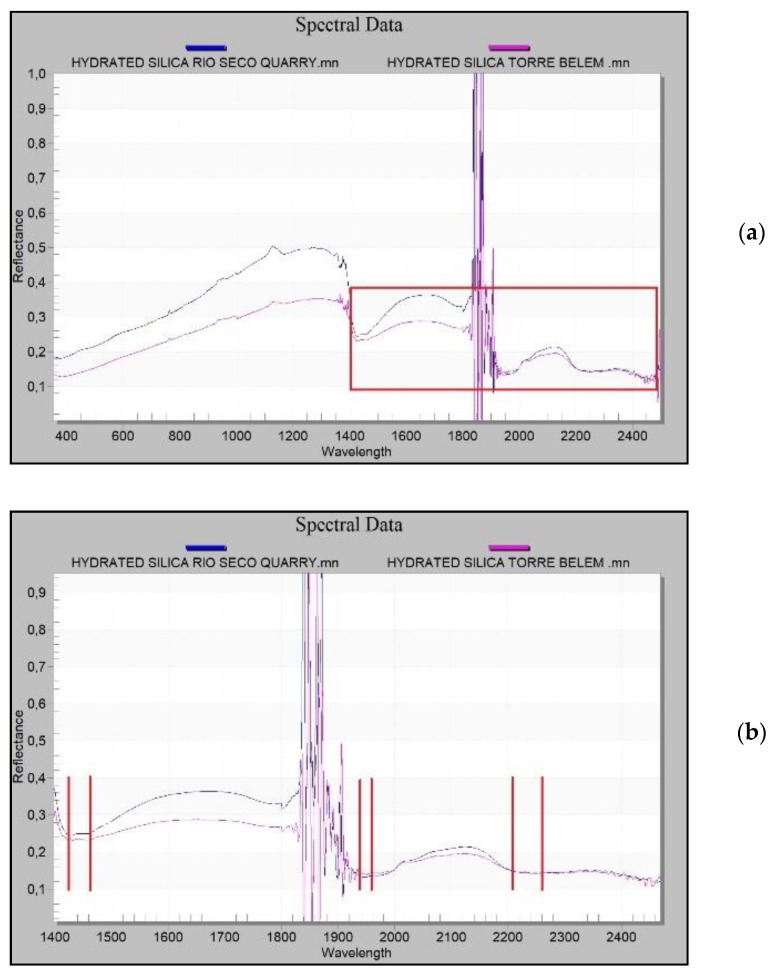
Comparison between the samples from the Tower of Belém and from the area of hydrated silica in the Rio Seco quarry. (**a**) Spectral signatures of the studied silica; (**b**) Common and characteristic absorption peaks of the silica types analyzed.

The silica measured in the ashlar of the Tower of Belém and the silica at the Rio Seco quarry have very similar reflectance curves. The silica from the quarry has a little more reflectance due eventually to both a different type of fracture and to erosion. This is not significant since absorbance and reflectance peaks are found at the same wavelengths (Figure 27a).

Enlarging the area of interest to include a spectral analysis of the silica (Figure 27a), it can be seen that the curves share the same wavelengths at the absorption peaks (Figure 27b). These values confirm this to be a hydrated cryptocrystalline kind of silica spectrally situated between the values for opal and calcedony. The absorption peaks for the groups Hydroxide (OH), Water (H2O) and Silicon Hydroxide (Si-OH), which define these hydrated types [67] are found at the same wavelengths in both cases studied (Tower of Belém and Rio Seco) (Figure 27b).

## 6. Conclusions

The documentation methodology used (Figure 3) is suitable for the detailed surveying of a heritage building, as is the case of the Tower of Belém. To carry out a project of this type, the data acquisition and processing time must be optimized based on the necessary resolution. Although each historical heritage monument has its own distinctive construction characteristics, it is true that there are many elements that are applicable as a methodology to other monuments. This particular case raised the challenge of collecting spatial data from the walls facing the river. The use of close-range photogrammetry from images taken from a vessel in movement at a constant speed provided an adequate solution to the problem. All scans were within the same coordinate system (ETRS89 PT-TM06) in order to register non-adjacent TLS point clouds and to register TLS and close-range photogrammetry point clouds. The use of, at least, four control points and one or two check points determined by GNSS to georeference the clouds acquired with TLS met the needs of the project in spite of the precision being inferior to that from the survey itself. Regarding documentation by close-range photogrammetry, the usual way to georeference by bundle adjustment using control points determined with a total station, yielded worse results, with respect to overall point residuals, than when those control points were picked up from the TLS point cloud, although the RMS values on control and check points were better. The only approach that yielded a RMS value near the absolute precision of the georeferenced TLS point clouds and behaved also good in the overall residuals, measured in a set of about 50,000 points, was the alignment of the photogrammetric cloud to the reference TLS cloud by the ICP algorithm.

The root mean square error in the adjustment of the TLS point clouds has been less than ±1.5 cm (between ±1.1 and ±1.35 cm at the control points and between ±0.91 and ±1.01 cm at the check points). On the other hand, in the areas in which it has not been possible to use TLS and it has been necessary to perform close-range photogrammetry in conditions of added difficulty from a boat or a very narrow place, the accuracies achieved have been about ± 2.7 cm on the exterior façade and ±1.2 cm on the spiral stairs.

To obtain a high-resolution 3D model, the TLS scans have been made with a 1/4 − 4× configuration with photographic capture to obtain the color of the point cloud. The geometry of the object to be surveyed determines the number of needed scans and the distance between positionings of the scanner. To obtain the necessary resolution, scans have always been made at distances < 25 m in large areas and < 8 m in narrow areas with spheres with diameter of 24 cm and 14.5 cm for registration of the TLS clouds.

As for the time required using each methodology, TLS took 70 h to acquire and 130 h to process using the SCENE software (Faro) [46]. In the photogrammetric survey of the exterior south wall, 15 min were necessary to acquire the images and 83 h to automatically process the 119 images, including the masking operation. The stairs required an estimated 8 h for the acquisition of over 1,000 images per section (not all included in processing) and a much longer period of estimated 300 h for processing and generation of the point clouds. This methodology is not the most effective for application to spiral stairs. A mobile laser scanner on a backpack carried by a person going up the stairs would certainly have been more effective, although this tool is not directly applicable and also poses challenges when it comes to data acquisition in objects that turn around themselves as the spiral stairs.

Regarding the techniques used in the study, the accuracy and versatility of the TLS versus the close-range photogrammetry in the acquisition of data in complex scenarios, such as the interior rooms of the tower, is evident. The capture of data from the south façade with TLS was considered unfeasible, even though its use at sea would be possible through the installation of stable platforms in the Tagus River. This was dismissed due to the high cost of necessary auxiliary means. Regarding the capture of the spiral staircase geometry, our TLS could not be used given the narrow space and number of necessary positions The Faro Focus 3D X330 scanner does not register a considerable area on the ground just under the equipment (the scanning vertical angle is 300° [51]) and the minimum scanning distance of 60 cm could not be achieved in 75 cm wide stairs after accounting for the space needed by the scanner in rotation.

The close-range photogrammetry is a more complex technique than TLS. Since it resorts to a passive sensor, exposure, aperture and sensibility must be optimal for obtaining sharp and luminous captures in all situations. If this is achieved, it significantly improves the calculation of homologous points and the correct location of the cameras in the photogrammetric model. The geometry of acquisition is also an important factor to achieve good accuracy in the photogrammetric model. In the case of the spiral staircase, the reduced physical space available to take the photographs, together with the available instruments, have been the main determining factors.

For the acquisition of the spiral staircase geometry, numerous resources have been used in terms of the number of images needed and the processing time. Data acquisition in these environments is complex due to the limited space for taking photographs, and for the distribution of control and check points. Other authors have used photographic systems at a higher cost than the used in this work, which provide greater agility in data collection, [47]. In this project, this element of the Tower has been photoreconstructed and georeferenced with low cost means (Sony α330 camera).

As for the origin of the stone, we conclude that the hypothesis presented in the discussion that the ashlars were taken from the Rio Seco quarry can be validated by the comparisons between the spectral signatures, which define, on one hand, the materials of the monument studied, and on the other, the materials from this quarry. Likewise, results show that the hydrated silica found in the ashlars of the Tower of Belém has a high probability of corresponding to those found at the Rio Seco quarry. This is supported by the spectroradiometric comparison between the surrounding rock of the nodule in the quarry (limestone) and the ashlars. This is also reinforced by the economic criterion of the original construction project owing to the shorter distance between the monument and this quarry.

The gray limestone ashlars were probably placed later due to the deterioration of the original material. The study confirms that these ashlars and the limestone of the Aqueduto das Águas Livres quarry belong to the same geological formation and that it is the same material, making it highly probable that the gray ashlars were extracted from this quarry. This raises the question of why a different colored limestone was used to that of the Lioz originally used in the construction of the monument, when the lighter colored stone was accessible from a nearer quarry. The old quarry of Rio Seco may have been exhausted, or the city might already have developed in the area surrounding the quarry at the time of the Tower’s restoration such that extracting the material was no longer feasible. Should the need arise to rebuild, rehabilitate or correct the monument in the future, this spectral study solves the question of where to take the material from in order to match the original stone. Therefore, in the event of a disaster (earthquake, tsunami, vandalism, etc.) with the results of this project, a detailed reconstruction of the Tower of Belém could be carried out, both geometrically and in terms of materials.

## Figures and Tables

**Table 1:** Configuration Parameters for the 3D TLS Survey with Focus 3D X330.

Surveyed Level (See Figure 6)	Number of Scans	Distance between Scanner Positions	Resolution/ Quality
NE	12	<25 m	1/4–4X
N-01	25	<10 m	1/5–3X
N00	23	<8 m	1/4–4X
N01	15	<10 m	1/4–4X
N02+NC	6+2	<8 m	1/4–4X
N03	8	<8 m	1/4–4X
N04	7	<8 m	1/4–4X
N05	10	<8 m	1/4–4X
N06	10	<8 m	1/4–4X

**Table 2:** Number of check and control points for scaling and georeferencing the point clouds.

Point Cloud Group	Total Number of Points	Control Points	Check Points
TLS. Scans group N-01 to N05	5	4	1
TLS. NE	6	4	2
TLS. N06	5	4	1
Close-range photogrammetry. Exterior walls	16	10	6

**Table 3:** RMS on control and check points for the bulwark exterior walls.

Approach	RMS [cm] at Control Points	RMS [cm] at Check Points
CA Identic point pairs and ICP	2.72 *	-
BA Points from total station	2.37	2.71
BA Points from TLS point cloud	4.96	6.88

* computed on 49999 points.

**Table 4:** Precision Obtained in the Orientation of Point Clouds.

Methodology	Section	Relative Precision	Absolute Precision
Terrestrial Laser Scanning	Cluster 1	0.65 cm	1.20 cm
	Cluster 2	0.15 cm	
	Cluster 3	0.39 cm	
Close-range photogrammetry	South Wall	0.39 pixel	2.72 cm
	Spiral stairs	-	1.22 cm

**Table 5:** Precision Obtained in Absolute Orientation. Control and Check Points.

Point Cloud Group	Control Points (cm)	Check Points (cm)
TLS. Scans group N-01 to N05	1.15	0.95
TLS. NE	1.35	1.01
TLS. N06	1.10	0.91
